# Prevalence of SCC*mec* types among Methicillin resistant Coagulase negative Staphylococci isolated from HIV patients in Chennai, South India

**DOI:** 10.1186/1471-2334-14-S3-P51

**Published:** 2014-05-27

**Authors:** Murugesan Saravanan, Betsy S Dass, Sankar Baby Abirami, Jaya Kumar Suriakumar, Padma Krishnan

**Affiliations:** 1 Dr. ALM PG IBMS, University of Madras, Taramani, Chennai, India; 2 Govt. Hospital of Thoracic Medicine, Tambaram, Chennai, India; 3 Institute of Microbiology, Madurai Medical College, Madurai, India

## Background

Methicillin resistant Coagulase negative staphylococci (MRCoNS) are opportunistic pathogens among HIV-AIDS patients and serve as a large reservoir of Staphylococcal Cassette Chromosome *mec* (SCC*mec*) that carries *mecA*. Very little is known about the prevalent SCC*mec* types among MRCoNS from HIV patients and its molecular epidemiology. Hence, this study was aimed at determining the distribution of SCC*mec* elements amongst MRCoNS isolates from HIV infected patients from Chennai, South India.

## Methods

52 clinically significant MRCoNS isolates from HIV patients were included in this study. Following speciation, isolates were subjected to antibiotic susceptibility testing and screening for methicillin resistance using cefoxitin disc (30µg) and confirmed by *mecA* gene PCR. The presence of SCC*mec* types (I - V) was determined using multiplex PCR.

## Results

Of the 52 isolates, *S. haemolyticus* (n =29, 56%) was predominant followed by *S. epidermidis* (n =13, 25%), *S. capitis* (n=4, 7%), *S. lugdunensis* (n =3, 6%) and *S. hominis*, (n =3, 6%). Highest resistance was shown towards ciprofloxacin (44%) followed by erythromycin (42%) and ofloxacin (39%). SCC*mec* typing revealed type I (n=26, 50%) to be predominant followed by type V (n=10, 19%), type III (n=7, 14%), type IV (n=4, 7%) and type I & V (n=2, 4%). Three (6%) isolates were nontypeable and type II was absent.

## Conclusion

The MRCoNS isolates carried genetically diverse SCC*mec* elements including types I, V, III, IV and I and V in combination with SCC*mec* type I being predominant among HIV patients, which may be attributed to majority of patients being hospitalized.

